# Cultural adaptation of the fear of cancer recurrence therapy (FORT) for Turkish breast cancer survivors

**DOI:** 10.1186/s40359-026-04915-6

**Published:** 2026-06-15

**Authors:** Aslı Eyrenci, Levent Ertuna, Ozan Bahcivan

**Affiliations:** 1 Maltepe University, İstanbul, Türkiye; 2 Sakarya University, Sakarya, Türkiye; 3 Oz Psychological Consultancy (Oz Psikolojik Danismanlik), Izmir, Türkiye

**Keywords:** Cultural adaptation study, FORT, Intervention, Fear of cancer recurrence, Ecological validity model

## Abstract

**Objective:**

Fear of cancer recurrence (FCR) is a common concern among breast cancer survivors in Turkiye. Despite its clinical relevance, culturally adapted interventions are scarce. This study aimed to adapt the Fear of Recurrence Therapy (FORT), a structured group-based intervention, for Turkish breast cancer survivors using the Ecological Validity Model (EVM).

**Methods:**

This study used a multi-phase mixed methods design conducted between February and September 2024. The cultural adaptation process included contextual literature review, translation and back-translation, ecological validity assessment by an expert panel (*n *= 6), cognitive interviews through focus groups with professionals and survivors (*n* = 6), and an adaptation workshop. A total of 12 participants contributed to the study, with no overlap between stages. Quantitative evaluations were analyzed using Gwet’s AC1 to assess inter-rater agreement across EVM domains. Qualitative feedback was analyzed through content analysis and used to guide manual revisions.

**Results:**

Gwet’s AC1 scores indicated moderate to very good agreement across sessions and components, especially in content, context, and metaphors. Revisions addressed technical language, culturally inappropriate metaphors (e.g., funeral imagery), and unclear therapeutic exercises. Visual and structural improvements included consistent formatting, color-coded infographics, and a glossary of key terms. Focus groups helped ensure linguistic clarity, emotional appropriateness, and cultural fit. The final six-session manual was rated highly acceptable by both experts and survivors.

**Conclusion:**

This study presents one of the first culturally adapted interventions targeting FCR in Turkiye. The participatory process emphasized ecological validity and cultural sensitivity, resulting in a clinically coherent and contextually appropriate manual. The adapted FORT program is now ready for pilot testing and provides a model for adapting evidence-based psycho-oncological interventions in other cultural settings.

**Supplementary Information:**

The online version contains supplementary material available at 10.1186/s40359-026-04915-6.

## Background

Cancer remains a major global health challenge, with rising incidence and mortality rates worldwide. However, advances in diagnostic technologies and access to healthcare have significantly improved survival rates and life expectancy among patients. As of 2022, there were an estimated 53.5 million cancer survivors globally [1], including over 470,000 in Turkiye [2]. Despite these improvements, many survivors continue to face substantial psychosocial challenges, with fear of cancer recurrence (FCR) being one of the most common and distressing concerns.

FCR -defined as the anxiety or concern that cancer may return or progress [3] -ranges from adaptive vigilance to clinically significant distress. While moderate FCR may motivate health-promoting behaviors, elevated levels are associated with chronic anxiety, impaired quality of life, and poor psychological adjustment [4–6]. Breast cancer survivors, particularly younger women and those with children, tend to report the highest FCR levels, influenced by both demographic factors and the concentration of research on this population [7]. Qualitative research further indicates that individuals experiencing high FCR often struggle with loneliness, intrusive thoughts, and uncertainty about the future, factors that can interfere with daily functioning and emotional well-being [8, 9]. These findings underscore the importance of accessible, targeted psychosocial interventions to manage FCR.

Several psychosocial interventions have been developed to reduce FCR among cancer survivors, including ConquerFear [10], SWORD [11], and Fear of Recurrence Therapy (FORT) [12]. Among these, FORT is a structured group-based intervention that integrates cognitive-behavioral and existential therapeutic components and has demonstrated feasibility and preliminary effectiveness in previous studies [12]. The intervention includes psychoeducation, cognitive restructuring targeting catastrophic thinking, relaxation techniques, and strategies aimed at increasing tolerance of uncertainty [12]. In addition to its therapeutic components, the group-based delivery format provides opportunities for peer support and shared experiences, which may further contribute to improved coping and emotional adjustment among survivors [13–15].

Despite growing international interest in interventions targeting FCR, psychosocial intervention research in Turkiye remains limited. Several studies conducted in Turkiye have examined FCR among cancer survivors, particularly among women with breast cancer, primarily focusing on psychological experiences, coping styles, and correlates of recurrence-related fears using qualitative or cross-sectional designs. However, intervention research specifically addressing recurrence-related fears remains scarce in the Turkish context. Existing psychosocial interventions for cancer survivors in Turkiye have generally focused on broader psychological distress, empowerment, or quality of life outcomes rather than targeting FCR directly [16–18]. This gap highlights the need for culturally adapted interventions specifically designed to address fear of cancer recurrence.

Yet intervention effectiveness depends heavily on cultural relevance, as beliefs, norms, and coping styles shape how individuals experience cancer-related fears. Without adaptation, interventions risk being ineffective or poorly received [19–21].

Cultural adaptation frameworks emphasize modifying intervention materials and delivery strategies while preserving their core therapeutic mechanisms. One widely used framework for culturally adapting psychosocial interventions is Bernal’s Ecological Validity Model (EVM) [22], which proposes several domains that should be considered when adapting interventions to new cultural contexts, including language, persons, metaphors, content, concepts, goals, methods, and context. By systematically examining these domains, researchers can maintain the theoretical foundations of an intervention while ensuring that culturally sensitive elements such as metaphors, examples, emotional expressions, and therapeutic exercises are meaningful and appropriate for the target population.

Accordingly, the present study aimed to culturally adapt the FORT program [12] for Turkish breast cancer survivors using the EVM [22] as the primary conceptual framework. The adaptation process followed a multi-phase design involving contextual review of the literature, translation and linguistic adaptation of the intervention materials, ecological validity assessment with stakeholders, cognitive interviews, and iterative revisions of the intervention manual. Through this systematic process, the study sought to ensure that the adapted intervention remained theoretically faithful to the original FORT model while being linguistically, culturally, and emotionally appropriate for Turkish cancer survivors. Ultimately, this work aims to address a critical gap in psycho-oncology services in Turkiye and to provide a culturally adapted intervention that can be evaluated in future randomized controlled trials (RCTs).

## Study design & cultural adaptation framework

This study employed a multi-phase cultural adaptation design to adapt the Fear of Recurrence Therapy (FORT) intervention for Turkish breast cancer survivors. The adaptation process was conceptually guided by Bernal’s Ecological Validity Model (EVM), which provides a framework for culturally adapting psychosocial interventions by examining culturally relevant domains such as language, metaphors, persons, concepts, content, context, goals, and methods [22]. These domains enable researchers to identify culturally sensitive elements of an intervention while preserving its theoretical foundations and core therapeutic mechanisms.

In addition, the adaptation process was informed by cultural adaptation frameworks for psychosocial interventions, particularly the model proposed by Barrera and Castro [23], which emphasizes iterative stakeholder involvement, contextual modification of intervention materials, and preservation of core intervention components during adaptation. Using these frameworks together allowed the research team to systematically evaluate the cultural relevance of the intervention while maintaining fidelity to the original FORT model.

The linguistic translation of the patient workbook followed the World Health Organization (WHO) recommendations for translation and back-translation of research instruments [24]. Importantly, these procedures were used only to guide the linguistic translation of the intervention materials and were not applied as the conceptual framework for the overall intervention adaptation process. The methodological quality of the study was evaluated against the criteria of the Mixed Methods Appraisal Tool (MMAT), version 2018 [25]. The completed MMAT checklist is provided as supplementary material (Additional File 1).

### FORT intervention

The original FORT program is a manualized group intervention [12] that integrates Cognitive Behavioral Therapy (CBT) techniques, including cognitive restructuring, behavioral activation, guided imagery, and psychoeducation. It also incorporates elements of existential therapy [26, 27], such as exploring fears through worst-case scenarios (e.g., death) and addressing emotions while focusing on the present moment and revisiting life goals post-diagnosis.

The intervention is delivered in six structured sessions and is accompanied by a patient workbook that includes psychoeducational material, therapeutic exercises, worksheets, and examples designed to help participants engage with the intervention content and practice coping strategies outside the group sessions.

The original FORT patient workbook is a structured intervention manual of approximately 60 pages organized across six sessions. It includes psychoeducational texts, conceptual diagrams (e.g., models of fear of recurrence and avoidance–exposure processes), structured cognitive-behavioral worksheets (e.g., thought records, worry monitoring tables), behavioral exercises, guided imagery scripts, relaxation instructions, and visually supported activities designed to facilitate between-session practice and skill consolidation.

### Permissions and ethical approvals

Permission for the cultural adaptation of FORT was initially obtained on October 11, 2022, from Dr. Sophie Lebel, one of the original developers of the program. The study was conducted in accordance with the principles of the Declaration of Helsinki. Ethical approval was granted by the Maltepe University Human Research Ethics Committee (Decision No. 2023/15 − 11). All participants provided written informed consent prior to participation. Data collection took place between February and September 2024. The expert panel review was conducted from February to March 2024, and focus group interviews were held between May and September 2024.

### Cultural adaptation procedure

#### Stages of cultural adaptation

The cultural adaptation process consisted of several sequential but iterative stages designed to ensure linguistic accuracy, cultural relevance, and contextual appropriateness of the intervention materials. These stages included contextual review of the Turkish literature, translation and linguistic adaptation of the workbook, ecological validity assessment of the translated materials, cognitive interviews with stakeholders, and a final adaptation workshop to consolidate revisions.

##### Stage 1. Contextual review of the turkish literature

In the first stage, a contextual review of Turkish research on fear of cancer recurrence (FCR) was conducted to identify culturally relevant experiences, coping styles, and psychosocial needs of breast cancer survivors in Turkiye. The purpose of this review was not to provide a comprehensive systematic synthesis of the literature but to inform the cultural adaptation of the intervention materials by identifying culturally relevant themes and contextual factors that may influence how FCR is experienced and addressed in psychosocial interventions.

##### Stage 2. Translation and linguistic adaptation of the workbook

In the second stage, the FORT patient workbook was translated from English into Turkish using a hybrid translation approach combining AI-assisted translation tools (DeepL and ChatGPT) with manual revision by the research team. The translated materials were subsequently back-translated into English to assess semantic and conceptual equivalence with the original version.

Discrepancies between the original and back-translated versions were discussed within the research team until consensus was reached. These translation procedures followed the WHO translation and back-translation recommendations, which were applied specifically to ensure linguistic accuracy and conceptual equivalence between language versions.

##### Stage 3. Ecological validity assessment

To evaluate the cultural appropriateness of the translated workbook, the materials were assessed according to the domains of the EVM (see Table 1).

**Table 1:** Ecological validity model components

Components	Description
Language	Clear and understandable language for breast cancer survivors in Turkiye.
People	Content should reflect ethnic and class differences within the breast cancer survivor population in Turkiye.
Metaphors	The use of culturally appropriate metaphors, discourses, and symbols that resonate with Turkish breast cancer patients.
Content	The content should align with the cultural values, traditions, and customs of Turkish breast cancer survivors.
Concepts	The intervention components must be culturally relevant to Turkish breast cancer survivors.
Objectives	Consistent with the needs of breast cancer survivors, specifically addressing fear of recurrence.
Method	Methods used should be suitable for the cultural context of Turkish breast cancer survivors.
Context	The content must consider the psychological, sociopolitical, and economic conditions in which Turkish survivors live.

Six advisory experts -including three mental health professionals and three breast cancer survivors- reviewed the translated materials and evaluated them using a Likert-type assessment form focusing on EVM components such as language clarity, conceptual relevance, metaphors, and contextual appropriateness.

The panel size of six experts (three professionals and three survivors) is consistent with recommendations for content validity and cultural adaptation studies, where panels of five to ten evaluators are typically considered sufficient to provide meaningful agreement estimates and identify culturally problematic content [28, 29]. In cultural adaptation research, the primary goal of expert review is to obtain targeted, in-depth evaluations from individuals with specific domain expertise rather than to achieve statistical generalizability. Similar panel sizes have been reported in comparable adaptation studies of psychological interventions [30, 31].

Advisory experts were recruited using convenience sampling. Eligible mental health professionals held a post-graduate degree in Clinical Psychology, had knowledge of cognitive behavioral therapy, had at least one year of experience working with breast cancer patients, and were fluent in both Turkish and English. Breast cancer survivors were eligible if they were over 18 years of age, had completed primary cancer treatment, and had access to an internet-connected device.

Experts were contacted through the Psycho-Oncological Association (TPOD), Oz Psychological Consultancy, and the researchers’ professional networks. Participants received the translated handbook and an evaluation form assessing EVM components and were given one month to provide feedback.

Quantitative ratings were later used to assess agreement across reviewers, while qualitative feedback helped identify culturally inappropriate expressions, unclear instructions, and problematic metaphors.

##### Stage 4. Cognitive interviewing Focus groups

In the cognitive interviewing stage, focus group discussions were conducted with mental health professionals and breast cancer survivors to further evaluate the comprehensibility, emotional appropriateness, and usability of the translated intervention materials.

Participants were recruited using convenience sampling. Eligibility criteria for professionals included a post-graduate degree in Clinical Psychology, knowledge of CBT, and experience working with breast cancer survivors. Survivors were eligible if they had completed primary treatment (chemotherapy, radiotherapy, and/or surgery) and had access to an internet-connected device. Access to an internet-connected device was required because the focus group discussions were conducted online via the Zoom platform. This requirement was therefore related to the data collection procedure rather than the intervention itself.

Two online focus group sessions (90 min each) were conducted via Zoom between May and September 2024. Each group included three participants: one group consisted of oncology professionals (e.g., psychiatrists, nurses, social workers) and the other of breast cancer survivors. These participants were independent from the six advisory experts who had participated in the ecological validity assessment, bringing the total number of participants across all stages to 12.

Although three participants per group is smaller than the typical range of four to eight recommended for conventional focus groups [32], this size was a deliberate methodological choice suited to the study’s objectives. The cognitive interviews aimed to obtain detailed, item-level feedback on specific workbook sections rather than to explore broad thematic patterns. Smaller groups allowed each participant sufficient time to examine and comment on individual exercises, metaphors, and instructions within the 90-minute sessions. This approach is consistent with recommendations for cognitive interviewing in instrument and material review, where small groups of three to five respondents per round are considered adequate for identifying comprehension problems and culturally inappropriate content [33]. In addition, multiple iterative sessions were conducted with each group until consensus was reached, which helped ensure that thematic coverage was not compromised by group size.

Sessions were audio-recorded with participants’ consent. During the discussions, participants evaluated sections of the workbook that had shown lower agreement in the previous stage and discussed issues related to clarity, cultural appropriateness, and emotional tone.

##### Stage 5. Adaptation workshop

In the final stage, an adaptation workshop was conducted to integrate feedback obtained from the ecological validity assessment and cognitive interviews. The research team reviewed the collected feedback and implemented final revisions to ensure that the adapted workbook remained theoretically consistent with the original intervention while being culturally appropriate for Turkish breast cancer survivors.

### Data analysis

#### Translation and review

A hybrid translation approach integrating AI-based tools and manual translation procedures was used to produce the Turkish version of the workbook. The translated materials were subsequently refined through iterative editing to ensure linguistic accuracy and cultural appropriateness.

As noted earlier, the translation procedures followed the WHO translation and back-translation recommendations to ensure semantic and conceptual equivalence. These procedures were used specifically for linguistic adaptation of the workbook and not as the primary framework for intervention adaptation.

Quantitative evaluations of EVM components were analyzed using Gwet’s AC1 coefficient to assess inter-rater agreement among advisory experts. The AC1 coefficient was selected over traditional kappa-based statistics (e.g., Cohen’s Kappa, Fleiss’ Kappa) for several reasons. First, kappa coefficients are susceptible to the “kappa paradox,” in which high observed agreement can yield misleadingly low kappa values when the distribution of ratings across categories is highly skewed [34, 35]. In cultural adaptation studies where expert reviewers tend to rate most items favorably, such marginal imbalance is common and can produce artificially deflated agreement estimates. Second, kappa’s chance-correction mechanism relies on marginal distributions to estimate expected agreement, making the statistic sensitive to trait prevalence rather than to actual rater disagreement [36, 37].Gwet’s AC1 addresses this limitation by using an alternative chance-agreement model that remains stable across varying prevalence conditions, providing a more accurate reflection of true inter-rater consensus. Third, simulation studies have shown that AC1 produces lower standard errors and more consistent estimates than Fleiss’ Kappa when the number of raters is small [36]. Given that the present study involved six raters evaluating culturally adapted materials on ordinal Likert-type scales with anticipated positive skewness, AC1 was considered the most appropriate agreement statistic for this design.

#### All statistical analyses were conducted using RStudio software with the irrCAC package

Qualitative feedback obtained from advisory experts and focus groups was analyzed using content analysis. Suggestions from participants were categorized according to EVM domains and used to guide revisions of the intervention manual.

#### Reporting of the cultural adaptation process

The reporting of the cultural adaptation procedures was informed by recommendations from the ADAPT framework for adapting interventions to new contexts and the RECAPT reporting guidelines for cultural adaptation of psychological interventions, which emphasize transparent reporting of adaptation decisions, stakeholder involvement, and contextual modifications made to intervention materials [38, 39].

## Results

### Contextual literature review findings

#### National studies on fear of cancer recurrence in Turkiye

The following findings summarize the contextual literature review conducted as Stage 1 of the cultural adaptation process. The purpose of this review was to identify existing evidence on fear of cancer recurrence in Turkiye in order to inform the cultural relevance of the intervention adaptation.

Recent studies conducted in Turkiye have increasingly examined FCR, particularly among breast cancer survivors who represent one of the most frequently studied survivor groups in FCR research. Research using qualitative, quantitative, and mixed-method designs has primarily focused on understanding survivors’ psychological experiences, coping strategies, and factors associated with recurrence-related fears rather than evaluating targeted interventions.

Qualitative studies have highlighted the complex emotional and existential dimensions of FCR among Turkish cancer survivors. For example, survivors often describe recurrence-related fears as persistent concerns associated with uncertainty about the future, fear of death, and worries about family responsibilities [17, 40]. These fears may be intensified by fatalistic beliefs and the perception that cancer recurrence is beyond personal control. Similarly, qualitative findings have shown that medical follow-up procedures and reminders of the disease may trigger recurrence-related anxieties and existential concerns among survivors [41].

Quantitative research has also identified several psychological and contextual factors associated with FCR. Studies conducted with Turkish cancer survivors have found that higher levels of fear are associated with maladaptive coping strategies, intolerance of uncertainty, and negative metacognitions [42, 43]. Conversely, protective factors such as psychological resilience and spiritual well-being have been associated with lower levels of FCR [44]. Other research has highlighted the potential influence of contextual factors such as socioeconomic status and patient–physician communication on recurrence-related fears [45, 46].

Despite the growing body of descriptive research on FCR in Turkiye, intervention studies specifically targeting recurrence-related fears remain scarce. Existing psychosocial intervention studies conducted with cancer survivors have primarily focused on broader psychological outcomes, such as distress, empowerment, or quality of life. For example, a psychoeducation program implemented with breast cancer survivors reported improvements in psychological well-being but did not specifically target FCR [17]. Similarly, a group-based psychoeducation and therapy program demonstrated reductions in psychological symptoms but did not include FCR-specific intervention components or measurement tools [16]. In another randomized controlled trial, an empowerment-based intervention improved resilience and posttraumatic growth among cancer survivors, although the program was not designed specifically to address fear of recurrence [18].

Overall, the existing literature indicates that fear of cancer recurrence is a common and distressing psychological concern among Turkish cancer survivors, particularly among women with breast cancer. However, the absence of culturally tailored psychosocial interventions specifically designed to address recurrence-related fears highlights an important gap in psycho-oncology services in Turkiye. The present cultural adaptation of the Fear of Recurrence Therapy (FORT) intervention was designed to address this gap by developing an intervention that is both theoretically grounded and culturally appropriate for Turkish cancer survivors.

### Ecological validity assessment findings

#### Translation

##### First translation

The FORT patient workbook was translated from English to Turkish by a bilingual research team, with the assistance of AI programs (DeepL, ChatGPT).

##### Back translation

The research team backtranslated the workbook and compared it with the original to assess semantic, linguistic, and conceptual compatibility. Discrepancies were discussed until consensus was reached. The original authors were consulted for ambiguous terminology, particularly in the section on common thought errors. For instance, the error “I can’t stand it” was reworded after consulting with Christine Maheu, one of the authors, to better capture the intended meaning and cultural relevance. The final wording was approved by focus group participants.

#### Ecological validity assessment

The Turkish version of the FORT patient workbook was evaluated by professionals (*n* = 3) and breast cancer survivors (*n* = 3). Participants provided feedback based on the components of the EVM. Inter-rater agreement was calculated to evaluate the consistency of reviewers’ ratings regarding the cultural appropriateness of the translated workbook across the EVM domains. Thus, the agreement statistics reflect the convergence of reviewers’ evaluations rather than a comparison between the original and translated versions of the workbook.

As described in the Data Analysis section, Gwet’s AC1 was used to assess inter-rater agreement because the rating distributions showed positive skewness across most EVM components, a condition under which traditional kappa statistics may produce unstable estimates.

### Quantitative agreement across reviewers

Gwet’s AC1 scores (Table 2) indicated moderate to very good agreement across sessions, with Session 2 generally rated with lower consistency. For EVM components (Table 3), strong agreement was found across most domains. However, the “Language” component had weak agreement among professionals and moderate agreement among survivors.

**Table 2:** Gwet’s AC1 coefficient for the FORT patient handbook sections

Sections	Professionals	BC Survivors	Total
Introduction	0.83	0.91	0.87
Session 1	0.45	0.83	0.66
Session 2	0.57	0.60	0.59
Session 3	0.71	0.71	0.71
Session 4	0.80	0.83	0.82
Session 5	0.91	0.64	0.79
Session 6	1.00	1.00	1.00
All	0.68	0.71	0.72

*BC Survivors:* Breast Cancer Survivors

**Table 3:** Inter-rater agreement (Gwet’s AC1) across reviewers for the cultural evaluation of the FORT patient handbook sections

EVM Components	Professionals	BC Survivors	Total
Language	0.13	0.43	0.29
People	0.72	1.00	0.88
Metaphors	1.00	1.00	1.00
Content	0.90	0.81	0.85
Concepts	0.66	0.36	0.52
Objectives	1.00	1.00	1.00
Method	1.00	0.90	0.95
Context	1.00	1.00	1.00

### Qualitative feedback from advisory experts

Using a quick content analysis, we identified the key feedback, critiques, and suggested changes from advisory experts to tailor the intervention manual for the context of Turkish breast cancer survivors. We categorized feedback according to EVM components, as well as visual design and format:

- Language, concepts, and people: Participants raised concerns about the use of technical psychological terminology (e.g., “relapse,” “hypothesized,” “alternative”) and recommended the use of more accessible language or brief explanatory notes. Accordingly, the manual was revised to improve clarity and emotional accessibility by incorporating plain-language expressions and a glossary of key terms. For instance, the clinical term “relapse” was replaced with the more explanatory phrase “the disease returning” (hastalığın geri gelmesi). Similarly, abstract cognitive terminology such as “hypothesized thoughts” was reformulated as “assumed or possible explanations” (varsayılan düşünce / olası açıklama), and “alternative thoughts” were presented as “another way of looking at the situation” (başka bir bakış açısı) to enhance conceptual engagement. Moreover, rather than using the direct translation of “guided imagery” (yönlendirilmiş imgelem) -perceived by participants as technical and distant - the culturally more intuitive expression mental visualization (zihinsel canlandırma) was preferred. These modifications aimed to preserve therapeutic intent while increasing comprehensibility and emotional resonance.
- Metaphors and concepts: Feedback highlighted difficulties in understanding examples of cognitive distortions (e.g., catastrophizing, black-and-white thinking). Examples were revised or replaced with culturally relevant and comprehensible alternatives, particularly those related to cancer narratives.
- Methods and Objectives: Suggestions focused on simplifying complex diagrams (e.g., the FCR model), clarifying exercise instructions, and avoiding unintended interpretations (e.g., overly positive affirmations seen as “toxic positivity”).

### Cognitive interviewing findings

#### Focus group discussions (professionals & breast cancer survivors)

**Fig. 1:**
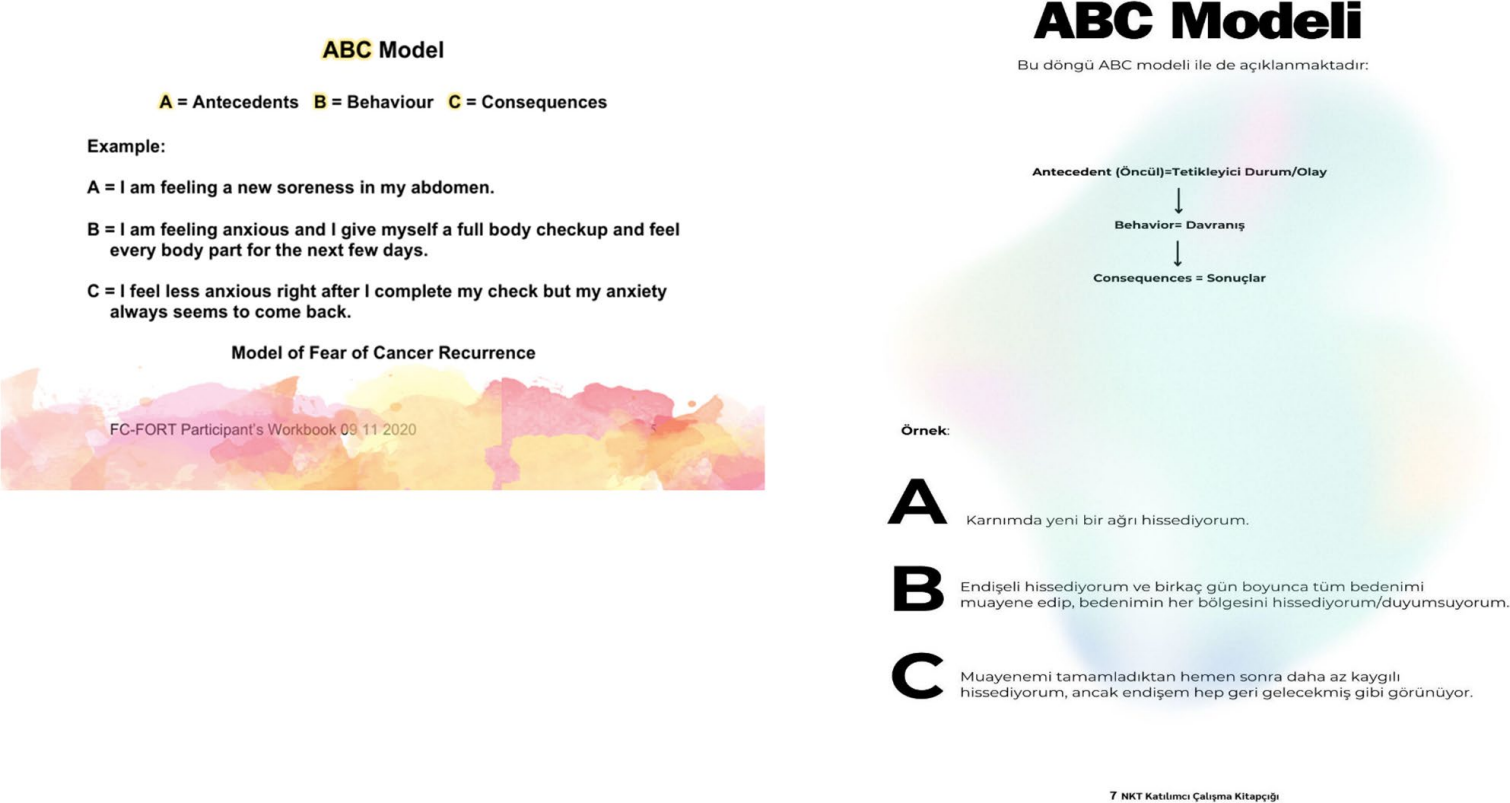
The ABC model in the original and culturally adapted manual

**Fig. 2:**
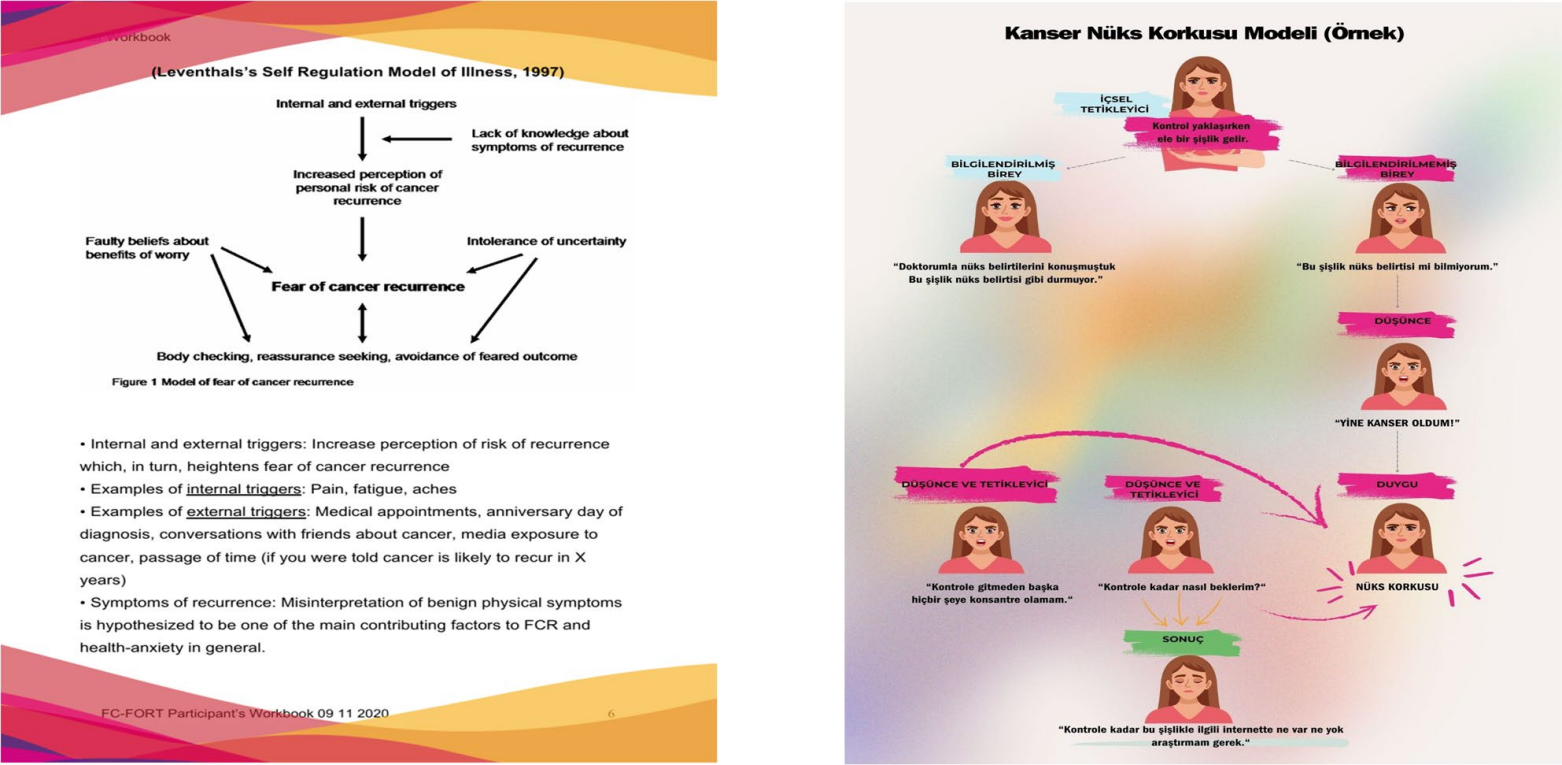
The FCR model in the original and adapted manual

Comprehensibility, relevance, and acceptability of the manual were evaluated during focus groups. Both professionals’ (*n* = 3) and survivors’ groups (*n* = 3) identified formal errors and linguistic inconsistencies, including shifts between active and passive voice and stylistic fragmentation. Passive constructions were standardized, and second-person pronouns (“you”) were adopted to create a warmer tone. The issues identified by participants for each section were noted and categorized by session, with corresponding changes made to the handbook based on their recommendations. Following these revisions, the final content was reviewed and approved by both professionals and survivor groups, incorporating feedback from a total of four focus groups. The suggestions and related revisions for each section are outlined below:

- Introduction: Professionals recommended adding a clear statement outlining the manual’s purpose and target audience. A brief introductory paragraph and a glossary of key terms were added to improve clarity and accessibility.
- Session 1 – Theoretical models: Participants found the presentation order of CBT, ABC, and FCR models confusing. While professionals suggested starting with the FCR model, the original sequence was retained to preserve conceptual logic. However, visual complexity was reduced using infographics and color coding. Clarifications were also made in the thought recording exercise (see Figs. 1 and 2).
- Cognitive distortions (e.g., personalization, filtering) were revised to better reflect the intended concepts and cultural context. Cancer-related and neutral examples were balanced to avoid emotional overload while maintaining relevance.
- Session 2 – General format and visual design: Participants criticized inconsistent and potentially triggering visuals (e.g., skulls, stick figures). In response, a full visual overhaul was undertaken to ensure stylistic consistency and emotional appropriateness, using soft tones and relatable imagery.
- Session 3 – “Why Worry” exercise and emotional language: The phrase “Everyone worries from time to time” was removed, as participants felt it undermined the therapeutic intent and prematurely normalized worry. This revision aimed to allow patients to explore their anxiety before reframing it.
- Session 4 – Emotional exposure: The suggestion to imagine one’s own funeral was deemed culturally inappropriate and potentially harmful in group settings. While addressing existential fears remains important, the specific example was omitted to avoid emotional distress, allowing participants to generate their own personally meaningful scenarios in a non-directive manner. Moreover, wording that seemed vague or emotionally directive (e.g., “your worst fear”) was replaced with more culturally acceptable and therapeutically appropriate alternatives (e.g., “your most overwhelming fear”). Certain terms like “guided imagery” were replaced with more familiar phrases (e.g., “mind visualization”).
- Session 5 – Coping with fear: Certain culturally sensitive content, such as imagining one’s funeral or writing a will, was deemed inappropriate for group settings. Initially, it was suggested that the content be reframed (e.g., preparing a will); however, this was rejected due to cultural discomfort with explicit references to death. The example was subsequently revised to focus on ‘refocusing on meaningful goals’ or ‘completing unfinished tasks,’ aligning with therapeutic objectives while respecting cultural sensitivities.
- Session 6 – Closure and summary: Professionals recommended adding a summary page to help participants reflect on progress. This was implemented using bullet points summarizing the six-session structure.

### Adaptation workshop & final revisions

At the final stage, an adaptation workshop was held to review and consolidate feedback from professionals’ and survivors’ focus groups. Using comparative summary tables the research team finalized revisions across content, language, visuals, and structure.

#### Manual design and layout

Significant updates were made to improve readability and emotional accessibility for participants.

- A clean, modern design was adopted using soft, low-saturation colors to reduce visual strain.
- Fonts were standardized (ITC Franklin Gothic for headings, Montserrat for body text) to enhance clarity and create visual contrast.
- The manual retained a vertical A4 format to facilitate easy printing and distribution.

#### Content enhancements

##### Introductory section

A brief explanation of the manual's purpose and use was added to orient participants.

##### Glossary

A glossary section was incorporated at the beginning of the workbook to enhance conceptual clarity and reduce the cognitive burden associated with technical psychological terminology. Terms like “relapse,” “inner speech,” “avoidance,” and “triggers” were explained in accessible language.

##### Session overviews

Linguistic inconsistencies resulting from the translation process were addressed, and textual coherence was enhanced by standardizing headings in the passive voice and adopting second-person pronouns to foster a more personal and empathetic tone.

#### Session-specific improvements

##### Session 1

Theoretical models were clarified using color-coded infographics. The thought recording form was exemplified, and cognitive distortion examples were revised to improve relevance and cultural fit.

##### Session 3

The “Why Worry” questionnaire was revised to eliminate potentially directive language; specifically, the phrase “Everyone may worry” was removed to maintain therapeutic neutrality and support open emotional exploration.

##### Session 5

The problematic example of “writing a will” was replaced with “reconnecting with meaningful goals or tasks previously postponed,” reflecting culturally appropriate coping strategies.

##### Session 6: 

A brief summary section was added at the end of the session to help participants reflect on what they had learned.

### Final adaptation decisions

All changes were reviewed and approved by the professionals’ and survivors’ groups. The finalized manual was deemed acceptable in terms of language, cultural relevance, and therapeutic coherence.

## Discussion

This study outlines the ecological and cultural adaptation of the FORT program for Turkish breast cancer survivors. Findings highlight the importance of cultural tailoring to ensure emotional safety, linguistic clarity, and relevance, core elements of ecological validity crucial for psycho-oncological group interventions. The use of the EVM provided a structured approach to identifying culturally sensitive elements of the intervention while preserving its theoretical foundations, consistent with recommendations from contemporary cultural adaptation frameworks for psychological interventions.

Culturally adapted interventions have consistently shown greater effectiveness compared to their non-adapted counterparts [19, 20]. Griner and Smith [21] reported a moderate effect size (d = 0.45) in a meta-analysis of such interventions. Over the past decade, numerous adaptation studies have emerged across various clinical populations and health domains [30, 31, 47, 48]. However, in Turkiye, adaptation efforts have primarily focused on psychometric tools [49–51], with relatively limited attention given to therapeutic programs [52, 53].

Our findings echo those of previous FORT adaptations conducted in Mexican and Spanish contexts [54, 55]. Participants raised concerns about abstract metaphors, intense exposure to content, and unfamiliar terminology. These challenges prompted key adaptations to improve clarity, emotional fit, and therapeutic relevance, especially for sensitive topics like death. For instance, references to wills or funerals were replaced with culturally appropriate themes like life goals and values. Our adaptation process aligns with a virtual adaptation model [56], which stresses iterative stakeholder input to maintain core elements while improving emotional accessibility. The use of Gwet’s AC1 coefficient for inter-rater agreement provided more stable estimates than would have been obtained with Fleiss’ Kappa, given the positively skewed rating distributions observed across most EVM components. Importantly, the adaptation was shaped through a participatory process that involved expert consultation, focus groups with professionals and breast cancer survivors, and an adaptation workshop among researchers. This iterative, multi-method design reflects the best global practices in mental health adaptation [38, 39] and strengthens both the ecological validity and real-world applicability of the resulting manual. Survivor input played a particularly valuable role in identifying emotionally discordant elements and suggesting contextually resonant alternatives.

A key issue in the adaptation process concerned how to address avoidance, which is a central maintaining factor of FCR, while ensuring cultural acceptability of emotionally sensitive content. In the original FORT intervention, avoidance is targeted through cognitive–existential exercises that encourage patients to approach feared scenarios, including thoughts related to illness progression or death. During the Turkish adaptation, certain examples used in the original manual (e.g., imagining one’s own funeral or writing a will) were perceived by participants as culturally inappropriate or excessively distressing in a group context. Importantly, these elements were not removed in a way that would weaken the therapeutic mechanism. Instead, the exercises were reframed to focus on confronting feared illness-related scenarios and uncertainties (e.g., worries about recurrence, unfinished life goals, or concerns about family), while allowing patients to identify personally meaningful fears rather than prescribing culturally sensitive death-related imagery. In practice, therapists still encourage participants to approach rather than avoid distressing thoughts by using graded emotional exposure, cognitive restructuring of catastrophic interpretations, and discussion of uncertainty about the future. Thus, the core therapeutic mechanism targeting avoidance was preserved, while the examples used to facilitate this process were culturally adjusted to ensure emotional safety and group acceptability.

Cultural adaptation inevitably requires balancing fidelity to the original intervention with responsiveness to the cultural context of the target population. In the present study, adaptations were designed to preserve the core therapeutic mechanisms of FORT -particularly the focus on confronting feared thoughts, reducing avoidance, and increasing tolerance of uncertainty- while modifying elements that could hinder engagement or emotional safety within the Turkish cultural context. Rather than altering the theoretical structure of the intervention, the adaptations primarily involved adjustments to language, metaphors, examples, and the framing of emotionally sensitive themes. This approach is consistent with recommendations from cultural adaptation frameworks, which emphasize maintaining intervention fidelity while modifying surface structures to improve cultural relevance and acceptability [22, 23, 38]. By preserving the underlying cognitive–existential processes of FORT while adapting culturally sensitive elements, the present study illustrates how evidence-based psycho-oncological interventions can be translated across cultural contexts without compromising their therapeutic integrity.

The document review highlighted a growing body of Turkish research on FCR, mostly descriptive or correlational in nature, with few intervention studies targeting recurrence-specific distress [40, 42, 57]. Existing psychoeducational or group therapy programs often lack structured cognitive-existential content, use non-specific outcome measures, or fail to address the sociocultural aspects of recurrence fear [17, 18]. Moreover, published intervention studies have rarely incorporated group-based formats tailored to breast cancer survivors, despite consistent evidence of elevated FCR in this population [40, 41].

These findings highlight the importance of tailoring both content and delivery. By targeting cognitive patterns like catastrophizing, boosting uncertainty tolerance, and using culturally resonant materials, the adapted FORT manual addresses a major gap in Turkish psycho-oncology literature and clinical practice. The absence of prior FCR-specific interventions underscores its significance.

Overall, the Turkish FORT adaptation shows how ecological and cultural factors can be integrated into evidence-based care without compromising effectiveness, offering a model for culturally grounded psycho-oncological practice.

### Clinical implications

The adapted FORT program offers a culturally sensitive model for supporting Turkish breast cancer survivors. By reducing emotionally intense content and simplifying abstract concepts, it fosters greater participant engagement and may help lower dropout rates in group settings. This process also underscores the importance of culturally competent therapist training and active survivor involvement, both of which can strengthen the therapeutic alliance and improve outcomes. Furthermore, the adapted manual serves as a valuable resource for psycho-oncology clinicians, providing culturally tailored scripts, exercises, and case examples that equip therapists with practical tools for addressing FCR in group settings. Integrating this program into oncology clinics and survivorship care plans could expand access to evidence-based psychosocial support across Turkiye.

### Strengths and limitations

This study has several strengths. It applied the Ecological Validity Model, combining qualitative and quantitative methods across diverse stakeholders. Gwet’s AC1 coefficient enhanced inter-rater reliability, and survivor input ensured culturally and emotionally relevant content. A limitation is the potential lack of generalizability across all Turkish subpopulations, as cultural values and language comprehension may vary by region, generation, or education level. The relatively small number of participants in the expert panel (*n* = 6) and focus groups (*n* = 3 per group) may also be seen as a limitation. However, the sample sizes were consistent with established practices in cultural adaptation and cognitive interviewing research, where the emphasis is on obtaining in-depth, expert-informed feedback rather than statistical representativeness [28, 33]. The iterative structure of the focus group sessions, which continued until consensus was reached, provided an additional safeguard for the adequacy of the qualitative data.

## Conclusion

This study presents the systematic adaptation of the FORT intervention for Turkish breast cancer survivors, guided by Bernal’s Ecological Validity Model. Feedback from survivors and professionals informed key revisions to language, tone, visuals, and exposure content, enhancing cultural relevance while maintaining the intervention’s theoretical integrity. The final version was deemed highly acceptable and is now ready for empirical testing. As one of Turkiye’s first culturally adapted psycho-oncological group interventions, the adapted program, referred to as Nüks Korkusu Terapisi (NKT) in the Turkish context, addresses a critical gap and provides a model for future adaptations. Future research should evaluate the program’s effectiveness in reducing FCR and examine its scalability across diverse Turkish oncology settings to improve access to culturally competent psychosocial care.

## Supplementary Information

Additional file 1. MMAT 2018 Quality Appraisal Checklist (.docx). Completed Mixed Methods Appraisal Tool checklist for the present study.

## Data Availability

Data are available on request from the corresponding author.

## Abbreviations

ABC: Antecedent–Belief–Consequence Model
AC1: Agreement Coefficient 1 (Gwet’s statistic)
ADAPT: Adaptation Framework for Evidence–Informed Interventions
CBT: Cognitive Behavioral Therapy
EVM: Ecological Validity Model
FCR: Fear of Cancer Recurrence
FORT: Fear of Recurrence Therapy
MMAT: Mixed Methods Appraisal Tool
NKT: Nüks Korkusu Terapisi
RCT: Randomized Controlled Trial
RECAPT: Reporting Cultural Adaptation in Psychological Trials
TPOD: Psycho–Oncological Association of Turkiye
WHO: World Health Organization

## References

[1] Global cancer burden growing. amidst mounting need for services. https://www.who.int/news/item/01-02-2024-global-cancer-burden-growing--amidst-mounting-need-for-services. Accessed 17 Mar 2026. PMC11115397 38438207

[2] Sung H, Ferlay J, Siegel RL, Laversanne M, Soerjomataram I, Jemal A, et al. Global Cancer Statistics 2020: GLOBOCAN Estimates of Incidence and Mortality Worldwide for 36 Cancers in 185 Countries. CA Cancer J Clin. 2021;71:209–49. 10.3322/caac.21660. 33538338 10.3322/caac.21660

[3] Vickberg SMJ. The Concerns About Recurrence Scale (CARS): a systematic measure of women’s fears about the possibility of breast cancer recurrence. Ann Behav Med Publ Soc Behav Med. 2003;25:16–24. 10.1207/S15324796ABM2501_03. 10.1207/S15324796ABM2501_03 12581932

[4] Corter AL, Findlay M, Broom R, Porter D, Petrie KJ. Beliefs about medicine and illness are associated with fear of cancer recurrence in women taking adjuvant endocrine therapy for breast cancer. Br J Health Psychol. 2013;18:168–81. 23134580 10.1111/bjhp.12003

[5] Lebel S, Ozakinci G, Humphris G, Mutsaers B, Thewes B, Prins J, et al. From normal response to clinical problem: definition and clinical features of fear of cancer recurrence. Support Care Cancer. 2016;24:3265–8. 27169703 10.1007/s00520-016-3272-5

[6] Humphris GM, Rogers S, McNally D, Lee-Jones C, Brown J, Vaughan D. Fear of recurrence and possible cases of anxiety and depression in orofacial cancer patients. Int J Oral Maxillofac Surg. 2003;32:486–91. 14759106

[7] Simard S, Savard J. Screening and comorbidity of clinical levels of fear of cancer recurrence. J Cancer Surviv. 2015;9:481–91. 25603948 10.1007/s11764-015-0424-4

[8] Johnson Vickberg SM, Duhamel KN, Smith MY, Manne SL, Winkel G, Papadopoulos EB, et al. Global meaning and psychological adjustment among survivors of bone marrow transplant. Psychooncology. 2001;10:29–39. 10.1002/1099-1611(200101/02)10:1<29::aid-pon482>3.0.co;2-y. 10.1002/1099-1611(200101/02)10:1<29::aid-pon482>3.0.co;2-y 11180575

[9] Mutsaers B, Jones G, Rutkowski N, Tomei C, Séguin Leclair C, Petricone-Westwood D, et al. When fear of cancer recurrence becomes a clinical issue: a qualitative analysis of features associated with clinical fear of cancer recurrence. Support Care Cancer. 2016;24:4207–18. 10.1007/s00520-016-3248-5. 27169700 10.1007/s00520-016-3248-5

[10] Butow PN, Turner J, Gilchrist J, Sharpe L, Smith AB, Fardell JE, et al. Randomized Trial of ConquerFear: A Novel, Theoretically Based Psychosocial Intervention for Fear of Cancer Recurrence. J Clin Oncol. 2017;35:4066–77. 10.1200/JCO.2017.73.1257. 29095681 10.1200/JCO.2017.73.1257

[11] Van De Wal M, Thewes B, Gielissen M, Speckens A, Prins J. Efficacy of Blended Cognitive Behavior Therapy for High Fear of Recurrence in Breast, Prostate, and Colorectal Cancer Survivors: The SWORD Study, a Randomized Controlled Trial. J Clin Oncol. 2017;35:2173–83. 10.1200/JCO.2016.70.5301. 28471726 10.1200/JCO.2016.70.5301

[12] Lebel S, Maheu C, Lefebvre M, Secord S, Courbasson C, Singh M, et al. Addressing fear of cancer recurrence among women with cancer: a feasibility and preliminary outcome study. J Cancer Surviv. 2014;8:485–96. 10.1007/s11764-014-0357-3. 24756313 10.1007/s11764-014-0357-3

[13] Savard J, Savard M-H, Caplette-Gingras A, Casault L, Camateros C. Development and feasibility of a group cognitive-behavioral therapy for fear of cancer recurrence. Cogn Behav Pract. 2018;25(2):275–85. 10.1016/j.cbpra.2017.08.001.

[14] Classen CC, Spiegel D. Supportive-Expressive Group Psychotherapy. In: Watson M, Kissane DW, editors. Handbook of Psychotherapy in Cancer Care. 1st edition. Wiley; 2011. pp. 105–17. 10.1002/9780470975176.ch10

[15] Maheu C, Lebel S, Bernstein LJ, Courbasson C, Singh M, Ferguson SE, et al. Fear of cancer recurrence therapy (FORT): A randomized controlled trial. Health Psychol Off J Div Health Psychol Am Psychol Assoc. 2023;42:182–94. 10.1037/hea0001253. 10.1037/hea0001253 36862474

[16] Yavuzsen T, Karadibak D, Cehreli R, Dirioz M. Effect of group therapy on psychological symptoms and quality of life in Turkish patients with breast cancer. Asian Pac J Cancer Prev APJCP. 2012;13:5593–7. 10.7314/apjcp.2012.13.11.5593. 23317223 10.7314/apjcp.2012.13.11.5593

[17] Şengün İnan F, Üstün B. Fear of Recurrence in Turkish Breast Cancer Survivors: A Qualitative Study. J Transcult Nurs. 2019;30:146–53. 10.1177/1043659618771142. 29708040 10.1177/1043659618771142

[18] Üzar Özçetin YS. Hastalığı Aktif Dönemde Olmayan Kanser Hastalarına Uygulanan Güçlendirme Programının Travma Sonrası Büyüme Ve Psikolojik Sağlamlık Düzeylerine Etkisi. Doctoral dissertation. Hacettepe Üniversitesi; 2016.

[19] Benish SG, Quintana S, Wampold BE. Culturally adapted psychotherapy and the legitimacy of myth: a direct-comparison meta-analysis. J Couns Psychol. 2011;58:279–89. 10.1037/a0023626. 21604860 10.1037/a0023626

[20] Hall GCN, Ibaraki AY, Huang ER, Marti CN, Stice E. A Meta-Analysis of Cultural Adaptations of Psychological Interventions. Behav Ther. 2016;47:993–1014. 10.1016/j.beth.2016.09.005. 27993346 10.1016/j.beth.2016.09.005

[21] Griner D, Smith TB. Culturally adapted mental health intervention: A meta-analytic review. Psychotherapy. 2006;43:531–48. 10.1037/0033-3204.43.4.531. 22122142 10.1037/0033-3204.43.4.531

[22] Bernal G, Bonilla J, Bellido C. Ecological validity and cultural sensitivity for outcome research: issues for the cultural adaptation and development of psychosocial treatments with Hispanics. J Abnorm Child Psychol. 1995;23:67–82. 10.1007/BF01447045. 7759675 10.1007/BF01447045

[23] Barrera M Jr., Castro FG. A Heuristic Framework for the Cultural Adaptation of Interventions. Clin Psychol Sci Pract. 2006;13:311–6. 10.1111/j.1468-2850.2006.00043.x.

[24] Organization WH. Process of translation and adaptation of instruments. WHO. World Health Organization. 2017. https://www.who.int/substance_abuse/research_tools/translation/en/.

[25] Hong QN, Fàbregues S, Bartlett G, Boardman F, Cargo M, Dagenais P, et al. The Mixed Methods Appraisal Tool (MMAT) version 2018 for information professionals and researchers. Educ Inf. 2018;34:285–91. 10.3233/EFI-180221.

[26] Kissane DW, Bloch S, Smith GC, Miach P, Clarke DM, Ikin J, et al. Cognitive-existential group psychotherapy for women with primary breast cancer: A randomised controlled trial. Psychooncology. 2003;12:532–46. 10.1002/pon.683. 12923794 10.1002/pon.683

[27] Tomei C, Lebel S, Maheu C, Lefebvre M, Harris C. Examining the preliminary efficacy of an intervention for fear of cancer recurrence in female cancer survivors: a randomized controlled clinical trial pilot study. Support Care Cancer. 2018;26:2751–62. 10.1007/s00520-018-4097-1. 29500582 10.1007/s00520-018-4097-1

[28] Lynn MR. Determination and Quantification Of Content Validity. Nurs Res. 1986;35:382. 3640358

[29] Polit DF, Beck CT. The content validity index: Are you sure you know what’s being reported? critique and recommendations. Res Nurs Health. 2006;29:489–97. 10.1002/nur.20147. 16977646 10.1002/nur.20147

[30] Brown FL, Aoun M, Taha K, Steen F, Hansen P, Bird M, et al. The Cultural and Contextual Adaptation Process of an Intervention to Reduce Psychological Distress in Young Adolescents Living in Lebanon. Front Psychiatry. 2020;11. 10.3389/fpsyt.2020.00212. 10.3389/fpsyt.2020.00212 PMC7104812 32265759

[31] Sit HF, Ling R, Lam AIF, Chen W, Latkin CA, Hall BJ. The Cultural Adaptation of Step-by-Step: An Intervention to Address Depression Among Chinese Young Adults. Front Psychiatry. 2020;11. 10.3389/fpsyt.2020.00650. 10.3389/fpsyt.2020.00650 PMC7359726 32733296

[32] Krueger RA, Casey MA. Focus Groups: A Practical Guide for Applied Research. SAGE; 2014.

[33] Willis GB. Cognitive Interviewing: A Tool for Improving Questionnaire Design. SAGE; 2004.

[34] Feinstein AR, Cicchetti DV. High agreement but low Kappa: I. the problems of two paradoxes. J Clin Epidemiol. 1990;43:543–9. 10.1016/0895-4356(90)90158-L. 2348207 10.1016/0895-4356(90)90158-l

[35] Cicchetti DV, Feinstein AR. High agreement but low kappa: II. Resolving the paradoxes. J Clin Epidemiol. 1990;43:551–8. 10.1016/0895-4356(90)90159-M. 2189948 10.1016/0895-4356(90)90159-m

[36] Gwet KL. Handbook of inter-rater reliability: The definitive guide to measuring the extent of agreement among raters. Advanced Analytics, LLC; 2014.

[37] Gwet KL. Computing inter-rater reliability and its variance in the presence of high agreement. Br J Math Stat Psychol. 2008;61:29–48. 10.1348/000711006X126600. 18482474 10.1348/000711006X126600

[38] Movsisyan A, Arnold L, Evans R, Hallingberg B, Moore G, O’Cathain A, et al. Adapting evidence-informed complex population health interventions for new contexts: a systematic review of guidance. Implement Sci. 2019;14:105. 10.1186/s13012-019-0956-5. 31847920 10.1186/s13012-019-0956-5 PMC6918624

[39] Perera C, Salamanca-Sanabria A, Caballero-Bernal J, Feldman L, Hansen M, Bird M, et al. No implementation without cultural adaptation: a process for culturally adapting low-intensity psychological interventions in humanitarian settings. Confl Health. 2020;14:46. 10.1186/s13031-020-00290-0. 32684948 10.1186/s13031-020-00290-0 PMC7362525

[40] İzgü N. Post-Treatment Experiences of Breast Cancer Survivors: A Descriptive Phenomenological Study. Turk Klin J Nurs Sci. 2020;12:385–96. 10.5336/nurses.2019-73152.

[41] Üner FO, Korukcu O. A qualitative exploration of fear of cancer recurrence in Turkish cancer survivors who were referred for colposcopy. Health Soc Care Community. 2021;29:729–37. 10.1111/hsc.13326. 33662170 10.1111/hsc.13326

[42] Babadostu MK. Meme Kanseri Sonrası sağ Kalımlılarda Kanser Nüks Korkusu, Belirsizliğe Tahammülsüzlük, Üstbilişler ve Baş Etme Stratejileri Ilişkisinin Incelenmesi. Master’s Thesis. Maltepe University (Turkey); 2022.

[43] Oztas B, Ugurlu M, Kurt G. Fear of cancer recurrence and coping attitudes of breast cancer survivors. Eur J Cancer Care (Engl). 2022;31. 10.1111/ecc.13742. 10.1111/ecc.13742 36259514

[44] Koral L, Cirak Y. The relationships between fear of cancer recurrence, spiritual well-being and psychological resilience in non‐metastatic breast cancer survivors during the COVID‐19 outbreak. Psychooncology. 2021;30:1765–72. 10.1002/pon.5727. 33982371 10.1002/pon.5727 PMC8237000

[45] Alkan A, Yaşar A, Güç ZG, Gürbüz M, Başoğlu T, Sezgin Göksu S, et al. Worse patient–physician relationship is associated with more fear of cancer recurrence (Deimos Study): A study of the Palliative Care Working Committee of the Turkish Oncology Group (TOG). Eur J Cancer Care (Engl). 2020;29. 10.1111/ecc.13296. 10.1111/ecc.13296 32864838

[46] Geyikçeli E. Fear of cancer recurrence and examination of mental symptoms in individuals who have survived cancer [Master's thesis]. Çanakkale: Çanakkale Onsekiz Mart University, Graduate School of Health Sciences; 2023.

[47] Naeem F, Phiri P, Rathod S, Ayub M. Cultural adaptation of cognitive–behavioural therapy. BJPsych Adv. 2019;25:387–95. 10.1192/bja.2019.15.

[48] Salamanca-Sanabria A, Richards D, Timulak L, Castro-Camacho L, Mojica-Perilla M, Parra-Villa Y. Assessing the efficacy of a culturally adapted cognitive behavioural internet-delivered treatment for depression: protocol for a randomised controlled trial. BMC Psychiatry. 2018;18:53. 10.1186/s12888-018-1634-x. 29482586 10.1186/s12888-018-1634-x PMC5828178

[49] Kayihan H, Akel BS, Salar S, Huri M, Karahan S, Turker D, et al. Development of a Turkish Version of the Sensory Profile: Translation, Cross-Cultural Adaptation, and Psychometric Validation. Percept Mot Skills. 2015;120:971–86. 10.2466/08.27.PMS.120v17x8. 26057421 10.2466/08.27.PMS.120v17x8

[50] Mendi O, Yildirim N, Mendi B. Cross-cultural Adaptation, Reliability, and Validity of the Turkish Version of the Health Professionals Communication Skills Scale. Asian Nurs Res. 2020;14:312–9. 10.1016/j.anr.2020.09.003. 10.1016/j.anr.2020.09.003 32937201

[51] Telci EA, Aslan UB, Yagci N, Cavlak U, Kabul EG, Kara G, et al. The Turkish version of the Neck Bournemouth Questionnaire in patients with chronic neck pain: a cultural adaptation, reliability, and validity study. Arch Med Sci AMS. 2019;17:708. 34025841 10.5114/aoms.2019.89322 PMC8130469

[52] Acarturk ZC, Alyanak B, Cetinkaya M, Gulen B, Jalal B, Hinton DE. Adaptation of Transdiagnostic CBT for Turkish Adolescents: Examples From Culturally Adapted Multiplex CBT. Cogn Behav Pract. 2019;26:688–700. 10.1016/j.cbpra.2019.02.007. 10.1037/ort0000310 29345479

[53] Boran P, Dönmez M, Atif N, Nisar A, Barış E, Us MC, et al. Adaptation and integration of the thinking healthy programme into pregnancy schools in Istanbul, Turkey. BMC Pregnancy Childbirth. 2023;23:245. 10.1186/s12884-023-05572-y. 37046237 10.1186/s12884-023-05572-y PMC10091323

[54] Rivera Olvera I. Adaptación cultural de la terapia para reducir el miedo a la recurrencia (FORT) de supervivientes de cáncer de mama mexicanas. 2023.

[55] Estapé T, Gondón N, Gálvez-Hernandez CL, Rivera-Olvera I, Lebel S, Estapé J et al. Validación ecológica del programa FORT (Fear of Cancer Recurrence Therapy): una adaptación del español mexicano al español castellano: reporte preliminar. Psicooncología Pozuelo Alarcón. 2023;20(2):255–65. 10.5209/psic.91525.

[56] Lamarche J, Cusson A, Nissim R, Avery J, Wong J, Maheu C, et al. It’s time to address fear of cancer recurrence in family caregivers: usability study of an virtual version of the Family Caregiver—Fear Of Recurrence Therapy (FC-FORT). Front Digit Health. 2023;5:1129536. 10.3389/fdgth.2023.1129536. 37671170 10.3389/fdgth.2023.1129536 PMC10475944

[57] Zorba Bahçeli̇ P, Yazicioğlu Küçük B. Fear of Cancer Recurrence in Women with Breast Cancer: A Cross-Sectional Study after Mastectomy. Med Rec. 2022;4:315–20. 10.37990/medr.1094338.

