# Supplementary material for: Cultural adaptation of the fear of cancer recurrence therapy (FORT) for Turkish breast cancer survivors
Source: BMC Psychol. 2026 Jun 15;14:1078. doi: 10.1186/s40359-026-04915-6 (PMC13386598; doi:10.1186/s40359-026-04915-6)
Supplement: Supplementary file 1 — Additional file 1. MMAT 2018 Quality Appraisal Checklist (.docx). Completed Mixed Methods Appraisal Tool checklist for the present study. [file 40359_2026_4915_MOESM1_ESM.docx]

**Additional File 1. MMAT 2018 Quality Appraisal Checklist**

**Study:** Cultural Adaptation of the Fear of Cancer Recurrence Therapy (FORT) for Turkish Breast Cancer Survivors

**Reference:** Hong, Q. N., Pluye, P., Fàbregues, S., et al. (2018). Mixed Methods Appraisal Tool (MMAT), version 2018.

**SCREENING QUESTIONS**

**S1. Are there clear research questions?**

Yes. The study aimed to culturally adapt the FORT intervention for Turkish breast cancer survivors using the EVM.

**S2. Do the collected data allow to address the research questions?**

Yes. Quantitative (Likert-type ratings) and qualitative (content analysis of expert and survivor feedback) data were collected.

**CATEGORY 1: QUALITATIVE**

**1.1. Is the qualitative approach appropriate?**

Yes. Content analysis was used to identify culturally inappropriate elements and guide manual revisions.

**1.2. Are the qualitative data collection methods adequate?**

Yes. Audio-recorded focus groups and written expert evaluations were used.

**1.3. Are the findings adequately derived from the data?**

Yes. Feedback was categorized by EVM domains and linked to specific revisions.

**1.4. Is the interpretation of results sufficiently substantiated by data?**

Yes. Adaptation decisions were grounded in documented participant feedback.

**1.5. Is there coherence between data sources, collection, analysis, and interpretation?**

Yes. Sources (experts, survivors), methods (focus groups, written evaluations), and analysis (content analysis by EVM domains) were aligned.

**CATEGORY 4: QUANTITATIVE DESCRIPTIVE**

**4.1. Is the sampling strategy relevant?**

Yes. Convenience sampling recruited experts with specific domain knowledge (clinical psychology, CBT, breast cancer care).

**4.2. Is the sample representative of the target population?**

Can't tell. The sample was selected for domain expertise, not population representativeness. Acknowledged as a limitation.

**4.3. Are the measurements appropriate?**

Yes. Likert-type scales aligned with EVM domains; inter-rater agreement assessed using Gwet's AC1.

**4.4. Is the risk of nonresponse bias low?**

Yes. All six panel members completed their evaluations.

**4.5. Is the statistical analysis appropriate?**

Yes. Gwet's AC1 was selected with methodological justification provided in the Methods section.

**CATEGORY 5: MIXED METHODS**

**5.1. Is there an adequate rationale for using a mixed methods design?**

Yes. Quantitative ratings assessed agreement; qualitative feedback identified specific adaptation needs. Both were necessary.

**5.2. Are the different components effectively integrated?**

Yes. Low-agreement sections from quantitative analysis were prioritized for qualitative review in focus groups.

**5.3. Are the outputs of integration adequately interpreted?**

Yes. Agreement statistics and qualitative adaptation decisions are reported together by EVM domain.

**5.4. Are divergences and inconsistencies adequately addressed?**

Yes. Sections with weak quantitative agreement were examined further in focus groups; discrepancies between expert and survivor perspectives are discussed.

**5.5. Do the components adhere to quality criteria of each tradition?**

Yes. Qualitative: content analysis procedures. Quantitative: Gwet's AC1 with confidence intervals.
